# Oral cancer cells sustainedly infected with *Porphyromonas gingivalis* exhibit resistance to Taxol and have higher metastatic potential

**DOI:** 10.18632/oncotarget.16550

**Published:** 2017-03-24

**Authors:** Bok Hee Woo, Da Jeong Kim, Jeom Il Choi, Sung Jo Kim, Bong Soo Park, Jae Min Song, Ji Hye Lee, Hae Ryoun Park

**Affiliations:** ^1^ Department of Oral Pathology & BK21 PLUS Project, School of Dentistry, Pusan National University, Mulgeum-up, Yangsan 50612, South Korea; ^2^ Department of Periodontology, School of Dentistry, Pusan National University, Mulgeum-up, Yangsan 50612, South Korea; ^3^ Department of Oral Anatomy, School of Dentistry, Pusan National University, Mulgeum-up, Yangsan 50612, South Korea; ^4^ Department of Oral and Maxillofacial Surgery, School of Dentistry, Pusan National University, Mulgeum-up, Yangsan 50612, South Korea; ^5^ Institute of Translational Dental Sciences, Pusan National University, Mulgeum-up, Yangsan 50612, South Korea

**Keywords:** Porphyromonas gingivalis, oral cancer, Taxol, Notch1, metastasis

## Abstract

Major obstacles to improving the prognosis of patients with oral squamous cell carcinoma (OSCC) are the acquisition of resistance to chemotherapeutic agents and development of metastases. Recently, inflammatory signals are suggested to be one of the most important factors in modulating chemoresistance and establishing metastatic lesions. In addition, epidemiological studies have demonstrated that periodontitis, the most common chronic inflammatory condition of the oral cavity, is closely associated with oral cancer. However, a correlation between chronic periodontitis and chemoresistance/metastasis has not been well established. Herein, we will present our study on whether sustained infection with *Porphyromonas gingivalis*, a major pathogen of chronic periodontitis, could modify the response of OSCC cells to chemotherapeutic agents and their metastatic capability *in vivo*. Tumor xenografts composed of *P. gingivalis*–infected OSCC cells demonstrated a higher resistance to Taxol through Notch1 activation, as compared with uninfected cells. Furthermore, *P. gingivalis*–infected OSCC cells formed more metastatic foci in the lung than uninfected cells.

## INTRODUCTION

Oral squamous cell carcinoma (OSCC), which is the most common cancer of the oral cavity, is mainly treated with surgery alone. Because surgical treatment of OSCC sacrifices both important functionality and esthetics of the head and neck, surgical treatment of OSCC should be minimized or replaced with other treatment modalities such as chemotherapy and radiotherapy. However, chemotherapy is used as an adjuvant therapy, not as a single treatment modality, for OSCC because it rarely results in a cure and has demonstrated limited effectiveness in OSCC [1]. Therefore, improvement of OSCC responsiveness to chemotherapeutic agents is essential for patients with OSCC to obtain a better functional and cosmetic result from treatment that could be obtained through a deeper understanding of the reasons or mechanism involved in the low sensitivity of OSCC cells to chemotherapeutic agents.

Recently, the importance of signals from the tumor microenvironment that is composed of extracellular matrix, fibroblasts, and immune cells has been emphasized. Among the numerous factors in the microenvironment, signals of inflammatory stimuli confer resistance to chemotherapy and tumor progression [2, 3]. Accordingly, associations between inflammation and cancer cells have been extensively studied in a variety of cancers such as gastric, lung, and colorectal cancer, suggesting that inflammatory mediators are linked to therapeutic resistance and increased aggressiveness of tumor cells, including their invasive and metastatic abilities [3, 4]. It has been suggested that inflammation contributes to the formation, maintenance, and expansion of pluripotent cancer stem cells (CSCs), a small subpopulation of tumor cells with the capability of self-renewal, dedifferentiation, and tumorigenicity [5, 6]. Notably, CSCs are also thought to be a major reason why cancer cells demonstrate aggressive behaviors such as chemoresistance and metastasis. This hypothesis can be supported by numerous studies including observations that CSCs are expanded and that prostaglandin E2, an inflammatory mediator, induces CSC expansion and accelerates the formation of liver metastases in mice by activating NFκB [7, 8]. Therefore, it can be hypothesized that inflammatory signals from the microenvironment modulate the responsiveness of cancer cells to chemotherapeutic agents through the acquisition of stem cell properties.

Chronic periodontitis is one of the most common chronic inflammatory diseases of the oral cavity, especially in older patients. A relationship between periodontal diseases and oral cancer is strongly suspected because of the common occurrence of periodontitis, but this correlation has been investigated only in several epidemiological studies and a few experimental studies. The epidemiological studies demonstrated a higher risk of oral cancer in patients with periodontal disease [9–12], and experimental studies indicated that *Porphyromonas gingivalis*, a major pathogen involved in chronic periodontitis, increased the invasive capability of OSCC cells through the production of MMP-2 and 9 [13]. Recently, we also observed that acute and chronic exposure to *P. gingivalis* increased the migratory and invasive properties of oral cancer cells, with a higher secretion of MMPs [14]. In addition, it has been shown that prolonged and repeated chronic exposure to *P. gingivalis* helped provide OSCC cells with resistance to Taxol by promoting CSC properties, suggesting that chronic periodontitis plays a role in the chemoresistance of OSCC cells [15]. Although studies on a variety of cancers have been conducted for the association between cancer stemness and resistance to chemotherapy, there are few reports on a direct link between inflammation and chemoresistance. Furthermore, to our knowledge, no *in vivo* study has been performed on chronic periodontitis and the responsiveness of oral cancer to chemotherapeutic reagents. To clarify whether chronic periodontitis could modify the susceptibility of OSCC to chemotherapeutic agents *in vivo*, we examined whether prolonged and repeated *P. gingivalis* infection mimicking chronic periodontitis could affect the responsiveness of tumor xenografts to Taxol in mice. In addition, we investigated the mechanism involved in the chemoresistance of OSCC cells that were sustainedly infected with *P. gingivalis*, especially on the Notch pathway, because the most recent *in vitro* studies consistently suggested that Notch signaling promotes several malignant features of migration, invasion [16], chemoresistance [17], and stemness [18].

## RESULTS

### Slower *in vivo* tumor growth was exhibited by OSCC cells sustainedly infected with *P. gingivalis* than noninfected OSCC cells

**Figure 1:**
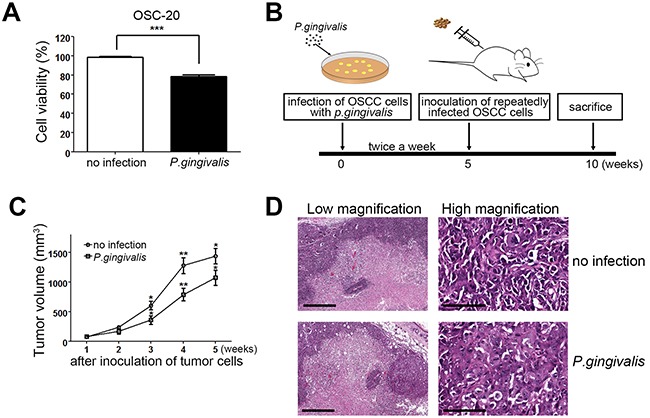
Tumor mass growth was slowed in OSCC cells with a sustained *P. gingivalis* infection **(A)** OSC-20 OSCC cells were infected with *P. gingivalis* at a MOI of 100 for 2 h and cultured for an additional 24 h. Relative growth rates of *P. gingivalis*-infected cells to non-infected cells were calculated based on a MTT assay. **(B)** To study the effect of *P. gingivalis* on tumor growth, non-infected and chronically infected OSC-20 cells with *P. gingivalis* were injected into the right and left flanks, respectively, of nude mice. **(C)** Tumor volume was measured once a week after subcutaneous implantation of tumor cells. Data are presented as mean ± standard deviation. Significance was assessed using a paired Student's *t* test. *, *P* < 0.05; **, *P* < 0.01. **(D)** Tumor masses involving *P. gingivalis*–infected or uninfected OSC-20 cells were excised, sectioned, and stained with H & **(E)** Representative images of H & E stained sections were obtained. Scale bars, 500 μm (left panels), 100 μm (right panels).

It has been shown that Ca9-22 and SCC25 OSCC cells infected with *P. gingivalis* showed lower proliferative activity than non-infected cells [19]. To further confirm that *P. gingivalis* infection contributes to the slower growth of OSCC cells, we infected OSC-20 OSCC cells with *P. gingivalis* twice and observed growth potential of *P. gingivalis*-infected and non-infected cells. As expected, *P. gingivalis*-infected OSC-20 cells displayed retarded growth in comparison with non-infected cells (Figure 1A). To investigate whether chronic periodontitis could affect the growth of oral cancer *in vivo*, we compared the tumor volume induced by sustainedly *P. gingivalis*-infected OSCC cells with that of uninfected control OSCC cells in nude mice. Briefly, cells were repeatedly infected with *P. gingivalis* twice a week for 5 weeks. Then the right flank of a mouse was injected with the infected OSC-20 cells, and the left flank of the same mouse was injected with uninfected OSC-20 cells. To rule out potential animal-to-animal variations, infected and uninfected tumor cells were simultaneously implanted in the same mouse (Figure 1B). Tumor volume was measured once a week, starting 8 days (1 w) after subcutaneous implantation of tumor cells. As we continued to monitor tumor growth during the following weeks, we were able to detect marked increases in tumor volume on both sides; tumors induced by uninfected control OSC-20 cells were significantly larger than those induced by *P. gingivalis*–infected cells (Figure 1C). The slower tumor growth observed with *P. gingivalis*–infected cells indicated that chronic infection with *P. gingivalis* could slow tumor growth in an OSCC xenograft mouse model. At Thirty-five days after inoculation, the tumor masses were excised, sectioned, and stained with H & E. These sections displayed histopathologic findings of OSCC, with prominent central necrosis (Figure 1D, left panels). At high magnification, the OSCC cells showed marked pleomorphism, little keratin production, and high mitotic activity, which are characteristics of moderately differentiated OSCC (Figure 1D, right panels). There was no definitive difference in histologic grade or morphological features between tumors induced by uninfected and *P. gingivalis*–infected OSC-20 cells.

### Sustained infection with *P. gingivalis* activated Notch1 in OSCC cells

**Figure 2:**
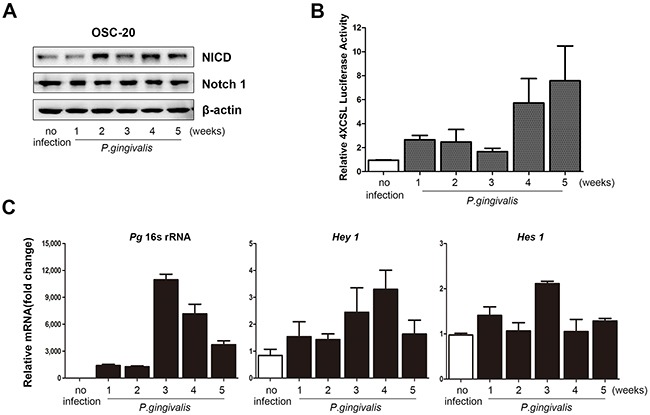
Notch1 was activated in OSCC cells with a sustained *P. gingivalis* infection **(A)** To mimic chronic infection with *P. gingivalis*, OSC-20 cells were repeatedly infected with *P. gingivalis* twice a week for the indicated periods. Notch1 and Notch intracellular domain (NICD) were analyzed using a Western blot analysis. **(B)** Cells were cotransfected with 4× CSL promoter-luciferase construct and a constitutively active *Renilla* luciferase plasmid. The graphs demonstrate relative luciferase activity. **(C)** The presence of *P. gingivalis* was confirmed by identifying 16S rRNA using real-time PCR. In addition, the levels of *Hey1* and *Hes1*, as well as downstream Notch targets, were investigated.

Our previous report demonstrated that prolonged and repeated infection of Ca9-22 OSCC cells with *P. gingivalis* induced CSC properties [15]. The Notch signaling pathway is known to play a critical role in maintaining the CSC population [20]. Thus, we investigated the expression of cleaved Notch1 (Notch intracellular domain, NICD), its active form, in OSC-20 cells chronically infected with *P. gingivalis*. As expected, protein levels of NICD were significantly increased over time in OSC-20 cells with a *P. gingivalis* infection (Figure 2A). In addition, increased levels of NICD in *P. gingivalis*–infected cells were observed in other types of OSCC cells lines (Supplementary Figure 1). Such activation of Notch1 in infected OSC-20 cells was further confirmed with significant stimulation of CSL promoter activity detected by a luciferase assay (Figure 2B), as well as changes in the mRNA levels of *Hes1* and *Hey1*, putative downstream targets of active Notch1 (Figure 2C, middle and right panels). With the presence of *P. gingivalis* within the OSCC cells that was verified by the expression of 16S rRNA in cell lysates (Figure 2C, left panel), these data indicate that *P. gingivalis* induced activation of Notch1 in OSCC cells.

### Sustained infection of OSCC cells with *P. gingivalis* caused resistance to Taxol through Notch1 activation

**Figure 3:**
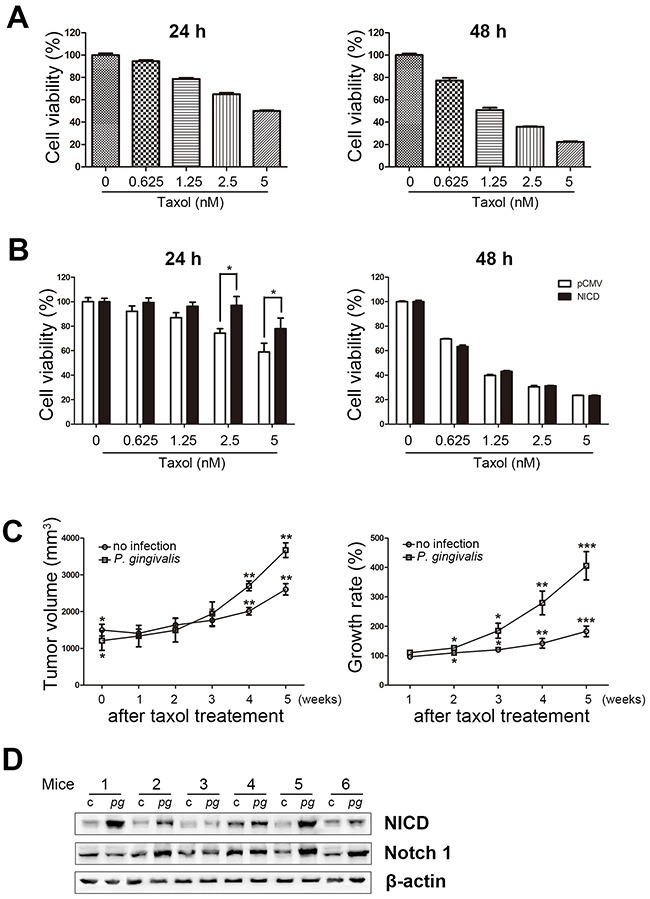
Overexpression of NICD decreases Taxol-induced cell death in OSCC cells **(A)** Cells were treated with various concentrations of Taxol (0, 0.625, 1.25, 2.5, and 5 nM) for 24 or 48 h and then subjected to an MTT assay. **(B)** Cell viability was analyzed in Taxol-exposed cells pretreated with or without NICD transfection. **(C)** OSC-20 cells with a sustained *P. gingivalis* infection and uninfected OSC-20 cells were implanted subcutaneously into the right and left flanks, respectively, of nude mice. Applications of Taxol (10 mg/kg) or vehicle through intraperitoneal injections were initiated 14 days after tumor inoculation and continued twice a week for a 4-week period. Data are presented as mean ± standard deviation. Significance was assessed using a paired Student's *t* test. *, *P* < 0.05; **, *P* < 0.01; ***, *P* < 0.001. **(D)** NICD expression was analyzed in tumor xenografts of *P. gingivalis*–infected OSCC cells using Western blot.

To determine the role of activated Notch1 with chemotherapy in oral cancers, the viability of NICD-overexpressing OSC-20 cells was assessed in the presence of Taxol, a well-characterized chemotherapeutic agent. When we first examined cellular toxicity to Taxol in naïve OSC-20 cells, their viability was gradually decreased in a dose-dependent manner at both 24 and 48 h of Taxol incubation (Figure 3A). Importantly, NICD-overexpressing OSC-20 cells exhibited higher viability than control cells transfected with an empty vector in the presence of Taxol. Such resistance to Taxol toxicity was prominent 24 h following a drug treatment, but not at a later time point (48 h) (Figure 3B). This is an interesting parallel to our previous finding that indicated reduced susceptibility to Taxol in Ca9-22 cells, another OSCC cell, upon chronic exposure to *P. gingivalis*. Moreover, these findings led to the hypothesis that *P. gingivalis* contributes to and induces activation of Notch1 that leads to the development of Taxol resistance in various OSCC cells.

To validate the idea that *P. gingivalis* induces Taxol resistance *in vivo*, a mouse xenograft model was established using OSC-20 cells. OSC-20 cells with a chronic *P. gingivalis* infection and uninfected OSC-20 cells were implanted subcutaneously into the right and left flanks, respectively, of nude mice. Application of Taxol (10 mg/kg) or vehicle via intraperitoneal injections was initiated 14 days after tumor inoculation and was continued twice a week for a 4-week period. When OSC-20–induced tumors were compared between *P. gingivalis*–infected and control xenografts following repeated Taxol treatment, we found a greater increase in size associated with *P. gingivalis* infection, albeit smaller initial measurements before application of Taxol (N = 6, *P* < 0.05; Figure 3C, left panel). In addition, the suppressive effect of Taxol on the growth rate of tumors was significantly greater in control xenografts, as compared with that of *P. gingivalis*–infected OSC-20 cells (Figure 3C, right panel), indicating lower sensitivity to Taxol in *P. gingivalis*–infected tumor xenografts. To examine whether an association could be established *in vivo* between the *P. gingivalis*–induced activation of Notch1 and chemoresistance demonstrated *in vitro* (Figure 2), we monitored the NICD levels in tumor xenografts with *P. gingivalis*–infected and control OSC-20 cells. Importantly, the level of NICD was significantly higher in *P. gingivalis*–infected tumor xenografts compared with uninfected controls (5 out of 6 pairs, Figure 3D), suggesting that the *P. gingivalis*–induced activation of Notch1 was responsible for the development of chemoresistance *in vivo*.

**Figure 4:**
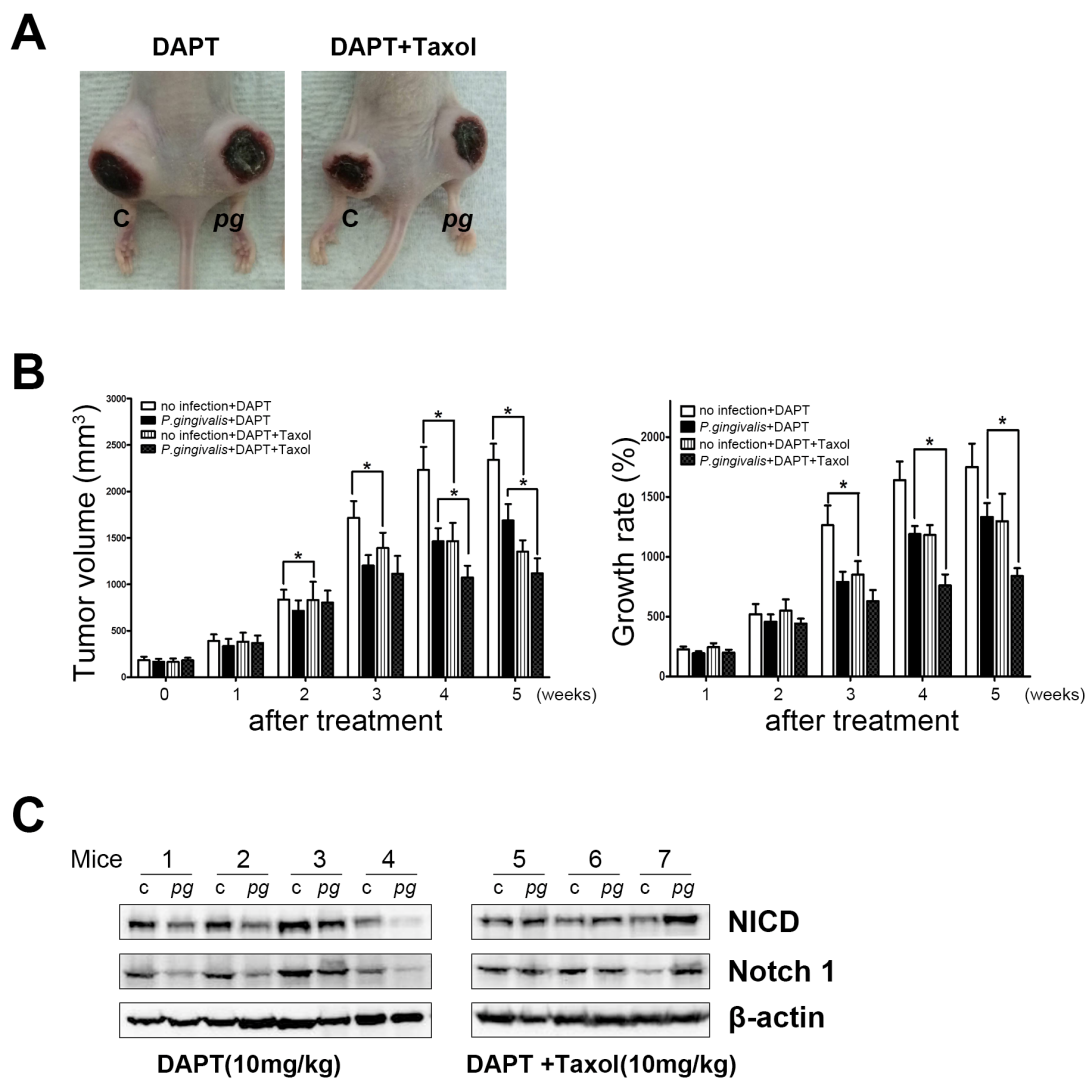
Notch inhibition reduces the chemoresistance of a *P. gingivalis*–infected OSCC xenograft Mice were treated with 10 mg/kg of DAPT with or without 5 nM of Taxol twice a week. **(A)** A representative photograph of mice treated with DAPT or DAPT–Taxol combination chemotherapy was obtained. **(B)** Tumor volume was measured once a week for 5 weeks. In addition, growth rates of tumor masses were compared and statistically analyzed. Data are presented as mean ± standard deviation. Significance was assessed using a paired Student's *t* test. *, *P* < 0.05. **(C)** A Western blot analysis indicated that DAPT treatment decreased NICD expression, and DAPT with Taxol induced no specific changes in NICD levels.

NICD-associated resistance to Taxol in *P. gingivalis*–infected tumors was further confirmed by co-application of DAPT, an inhibitor of Notch activation, as well as Taxol in a xenograft model (Figure 4A). In the presence of DAPT, the effect of Taxol as a tumor growth suppressor was clearly visible in both *P. gingivalis*–infected and uninfected xenografts (Figure 4B, left panel). However, as Taxol was repeatedly applied, a more prominent retardation of tumor growth was observed in *P. gingivalis*–infected xenografts (Figure 4B, right panel, *P* < 0.05). Enhanced sensitivity to Taxol following inhibition of *P. gingivalis*–induced Notch signaling was demonstrated to play a central role in the development of chemoresistance with chronic exposure of OSCC cells to *P. gingivalis*.

### Sustained infection with *P. gingivalis* increased the metastatic potential of OSCC cells

**Figure 5:**
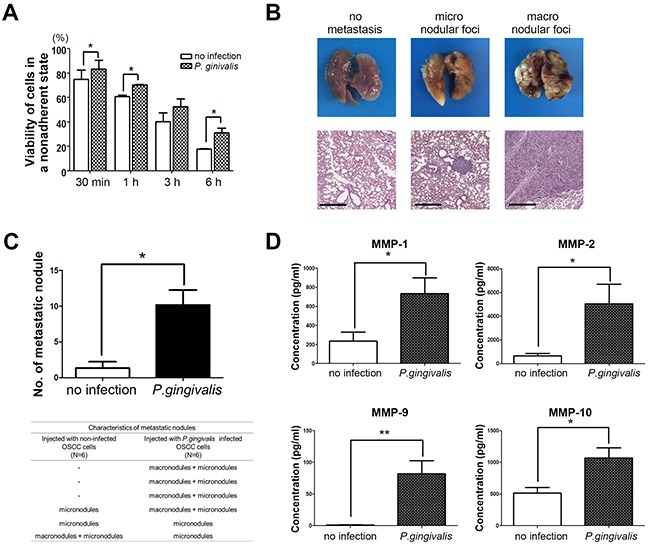
Sustained infection with *P. gingivalis* increased the pulmonary metastasis of OSCC *in vivo* **(A)** Viability of *P. gingivalis*–infected OSCC cells in nonadherent and agitated states was compared with uninfected cells. **(B)** Representative photographs of pulmonary metastatic foci were obtained at 30 days after intravenous injection of OSC-20 cells into the tail vein. Images of H & E sections were taken to display micronodular and macronodular metastatic lesions in lung tissues. Metastatic nodules which were grossly identified were considered macrodular foci. The nodules which were only validated under light microscopy were counted as micronodular foci, and the sizes of the micronodules were generally no more than 500 μm in diameter. Scale bars, 500 μm. **(C)** The number of metastatic nodules in lung tissue were counted and analyzed. In addition, characteristics of metastatic lung nodules from mice were investigated and compared depending on injected cell types. Data are presented as mean ± standard deviation. Significance was assessed using a paired or unpaired Student's *t* test. *, *P* < 0.05; **, *P* < 0.01. **(D)** Serum levels of human MMPs in mice injected with uninfected or *P. gingivalis*–infected OSC-20 cells were analyzed using a Luminex assay.

For cancer cells to establish a metastatic focus as a tumor mass, they travel a long distance within the blood stream. Therefore, cancer cells should survive in a movable and unattached state before their resettlement at metastatic sites such as the lungs, liver, or bones. We first examined the viability of OSC-20 OSCC cells with or without chronic exposure to *P. gingivalis* in a nonadherent and continuously agitated setting. The viability of both cell types decreased in a time-dependent manner, but *P. gingivalis*–infected OSC-20 cells were more resilient to such mechanical agitation, as compared with uninfected controls (Figure 5A, *P* < 0.05), suggesting that *P. gingivalis*–infected OSCC cells could withstand mechanical stress and survive within the blood stream, thus capable of forming metastatic foci.

To validate the idea that chronic *P. gingivalis* infection could promote metastasis through the blood stream *in vivo*, the metastatic abilities of uninfected and *P. gingivalis*–infected OSCC cells were compared in mice. Briefly, BALB/c mice were administered injections of OSC-20 cells (2 × 10^6^) into their tail vein and sacrificed 30 days later for removal of major organs including the lungs, liver, and kidneys to evaluate distant metastasis of OSC-20 cells. When metastatic lesions were detected, they were localized exclusively to the lung surface, sparing the liver and kidneys. The size of metastatic foci was variable, ranging from microscopic lesions that were not readily detected in gross specimens (Figure 5B, middle panels) to large nodules that replaced large portions of the lung lobes (Figure 5B, right panels). Indeed, the repetitive exposure of OSC-20 cells to *P. gingivalis* for 5 weeks resulted in metastatic foci in the lung tissues of all mice tested (six out of six). Importantly, four of six mice exhibited the formation of macronodules. In contrast, only one mouse injected with uninfected OSC-20 cells developed macronodules in the lungs. As a result, a fewer number of metastatic foci had formed in mice injected with uninfected OSC-20 cells than in mice injected with *P. gingivalis*–infected cells (Figure 5C). In addition to metastatic foci, the mice injected with *P. gingivalis*–infected OSC-20 cells showed higher serum levels of human MMPs including MMP-1, MMP-2, MMP-9, and MMP-10, in contrast to low to nearly undetectable MMPs in mice injected with uninfected OSC-20 cells (Figure 5D). Such low MMP levels may reflect the limited invasive ability of uninfected OSC-20 cells or alternatively reflect a reduced amount of MMPs released from only a few microscopic metastatic lesions.

## DISCUSSION

While acceleration of tumor growth by inflammation-driven mechanisms is well established [21], our understanding of tumor cell responses to microbial infection–induced inflammation can be complicated by multiple factors, including the types of host tumor cells and status of bacterial cells and/or host tumor cells. For example, previous studies have reported that live and heat-killed *P. gingivalis* infections promoted host cell survival and cellular apoptosis, respectively [22–24]. In other studies, live *P. gingivalis* induced apoptosis in trophoblasts but enhanced proliferation in gingival epithelial cells by modulating cell cycle progression [25, 26]. Considering these complex results, no clear conclusion has been drawn at this moment on the response of tumor cells to *P. gingivalis* infection.

There have been only a few *in vivo* studies that investigated the effect of *P. gingivalis* on tumor growth. According to one of these studies, growth of C26 colon carcinoma was accelerated by a *P. gingivalis* GroEL treatment in BALB/c mice [27]. Our previous *in vitro* analysis indicated that a transient *P. gingivalis* infection suppressed host cell proliferation in Ca9-22 OSCC cells by inducing G1 arrest and autophagy [19]. Furthermore, we have reported the reduced proliferation of Ca9-22 OSCC cells resulting from a prolonged and repeated infection with *P. gingivalis* that mimicked chronic inflammation [15]. A similar suppressive effect was also found with *P. gingivalis* in this study, as evidenced by reduced tumor growth in BALB/c xenograft mice harboring OSC-20 OSCC cells that were chronically infected with *P. gingivalis*. It is possible that soluble factors released from *P. gingivalis* may promote cell proliferation and contribute to tumor growth, but *P. gingivalis* itself may suppress proliferation of oral cancer cells and tumor mass growth, potentially by cell cycle arrest and autophagy, as we reported, or by residual immune reaction to signals from *P. gingivalis* in BALB/c mice.

In addition to its effect on tumor growth, our previous report demonstrated that prolonged and repeated infection with *P. gingivalis* induced epithelial mesenchymal transition and resistance to Taxol and slowed proliferation cycles in OSCC cells. The cells also displayed higher expression levels of CD44 and CD133, representative indicators for cancer stemness [15]. A number of signaling cascades, including the Notch1 pathway, have been recently linked to formation of CSCs, development of chemoresistance, and acquisition of an epithelial–mesenchymal transition (EMT) phenotype [28]. Our findings of enhanced NICD signaling in OSCC cells induced by chronic *P. gingivalis* infection (Figure 2), as well as decreased sensitivity of NICD-overexpressing OSCC cells to Taxol (Figure 3), suggest that chronic *P. gingivalis* infection plays an important role in allowing OSCC cells to develop chemoresistance in a Notch1-dependent manner. In addition, enhanced sensitivity of *P. gingivalis*–infected tumor xenografts to combinations of Taxol and DAPT, an inhibitor of Notch activation, further supports that the Notch signaling pathway has a central role in mediating the chemoresistance of chronically infected oral cancer cells.

Our results are in line with other studies that demonstrated the critical role of the Notch signaling pathway in the regulation of CSCs and chemoresistance [17, 18, 29]. Recent reports have also demonstrated the enhanced efficacy of chemotherapeutic drugs including docetaxel and paclitaxel when combined with DAPT in prostate cancer xenograft models and all platinum-resistant ovarian tumors, respectively [30–32]. While these findings suggest the need of combined Notch inhibition to improve efficacy of various chemotherapeutic reagents, whether a similar approach can be directly applied to target chronically inflamed cancer, especially periodontitis-associated oral cancer has never been examined. To our knowledge, our current results from both *in vitro* and *in vivo* analyses present the first experimental evidence supporting the usefulness of Notch inhibition in concert with conventional cytotoxic chemotherapy to effectively cure chronically inflamed OSCC in the presence of periodontitis-causing pathogens.

Experimental evidence from mouse cancer models and human cancer studies has demonstrated the tight association of chronic inflammation with tumorigenesis and tumor progression. However, our current understanding regarding inflammation in a metastatic cascade is mainly restricted to the effects of inflammatory mediators on metastasis, including TNF-α, IL-1 beta, and TGF-beta, as well as relevant molecular mechanisms [33]. A limited number of experimental studies have demonstrated the effect of bacterial infection on cancer metastasis with metastatic tumor growth promoted by the administration of bacterial LPS in mice [34–36]. It remains unknown whether bacterial pathogens such as *P. gingivalis* can directly influence the metastatic progression of oral cancer. A recent report indicated that a chronic infection involving *P. gingivalis/Fusobacterium nucleatum* through oral administration markedly enhanced the invasiveness of chemically induced OSCC in mice [37]. Herein, we presented the first experimental and molecular evidence demonstrating that OSCC combined with repetitive *P. gingivalis* infections resembling chronic periodontitis developed higher metastatic potential through the activation of the Notch signaling pathway, a key molecular player modulating the EMT and tumor angiogenesis. Thus, our findings may provide a rationale for a combined therapeutic approach in patients with oral cancer consisting of anti-inflammatory regimens and periodontitis treatments along with chemotherapy to decrease the pro-metastatic activity of chronically inflamed cancer cells.

In summary, we presented *in vitro* and *in vivo* experimental evidence supporting the idea that sustained infection with *P. gingivalis*, a major pathogen responsible for chronic periodontitis, promotes distant metastasis of oral cancer, as well as its resistance to anti-cancer agents. Our results suggest that resolution of chronic periodontitis may serve as a promising therapeutic target for metastatic and chemoresistant oral cancers.

## MATERIALS AND METHODS

### Cancer cell and bacterial cultures

The human OSCC cell line, OSC-20, was cultured in a 1:1 mixture of Dulbecco's Modified Eagle's medium and Ham's F-12 nutrient mixture (DME/F12; Hyclone, Logan, UT), supplemented with 10% fetal bovine serum (FBS; Atlas Biologicals, Fort Collins, CO), streptomycin (100 μg/mL), and amoxicillin and penicillin (100 IU/mL) (Gibco–Invitrogen Corporation, Rockville, MD) at 37 °C in a 5% CO_2_ incubator. The *P. gingivalis* strain, 381, was cultured anaerobically and grown in a GAM broth (Nissui, Tokyo, Japan) at 37 °C.

### Infection of OSCC cells with *P. gingivalis*

The OSCC cells were infected with a live *P. gingivalis* strain 381 at an MOI (multiplicity of infection) of 1:100 for 3 h at 37 °C in 5% CO_2_. Then, the cells were washed with PBS and cultured in fresh media until the next infection or harvest. Controls were subjected to the same media changes and wash conditions, without bacterial inoculation.

### Western blot analysis

Western blot analysis was performed according to our previous procedures [15, 19]. Briefly, approximately 40 μg of protein from each sample was denatured and then loaded in each lane of 10–12% PAGE gel. Subsequently, proteins were transferred onto a PDVF membrane and blocked for 1 h, incubated with primary antibodies overnight, and finally incubated with a horseradish peroxidase–conjugated secondary antibody (Santa Cruz Biotechnology, Santa Cruz, CA). The following primary antibodies were used: Notch1, Notch intracellular domain, and actin.

### RNA extraction and real-time PCR

Total RNA was extracted from cells using an RNeasy Mini kit (Qiagen, Hilden, Germany) according to the manufacturer's instructions. cDNA was synthesized, and bacterial 16S rRNA, along with the mRNAs of *Hes*1 and *Hey* and GAPDH, was detected using 2-step quantitative real-time PCR with a QuantiTect reverse transcription kit (Qiagen) and TOPreal SYBR Green PCR Kit (Enzynomics, Seoul, South Korea) in an ABI 7500 real-time PCR detection system (Applied Biosystems, Foster City, CA).

### Cell transfection

Subconfluent OSC-20 cells were seeded in 6-cm plates and transfected on the following day with 2 μg of plasmid DNA using a FuGENE 6 reagent (Roche Diagnostic Corp., Penzberg, Germany), according to the manufacturer's recommendations. The amount of transfected DNA was kept constant with the addition of appropriate amounts of the parental empty vectors.

### Luciferase assay

OSC-20 cells were seeded into 24-well plates, and 4× CSL reporter plasmids (0.5 μg each) were separately transfected into the cells. After 48 h, the cells were lysed in a passive lysis buffer; luciferase activity was measured with a Dual-Luciferase Reporter Assay System (Promega, Madison, WI); and target promoter-driven firefly luciferase activity was normalized to that of the *Renilla* control. Each experiment was repeated three times.

### MTT assay

The cells were treated for 24 h or 48 h with various concentrations of Taxol 24 h after transfection. Subsequently, the medium was removed, and the cells were incubated in an MTT solution (1 mg/mL; 100 μL/well) at 37°C for 4 h. The supernatant was then removed, and 100 μL of dimethyl sulfoxide (DMSO) was added to each well to dissolve the insoluble formazan. The 96-well plates were shaken for 10 min, and absorbance at 545 nm was measured using a microplate reader. Then, the percentage of cell growth was calculated based on 100% growth without treatment with Taxol.

### Cell viability in a nonadherent and agitated state

The cells were harvested and placed into a 15-mL polystyrene tube in the presence of cell media. The tubes were continuously shaken at 37 °C using a shaking incubator (200 rpm/min); then, the number of viable and nonviable cells was counted at the indicated time points.

### Luminex assay

Levels of matrix metalloproteinases (MMPs) in the serum of mice were measured using a Milliplex Map Human MMP panel 2 kit (R & D system, Minneapolis, MN). The Luminex 200 platform, coupled with BioRad Bio-Plex software (BioRad), was used to measure the MMP levels according to the manufacturer's protocols.

### Nude mouse xenograft chemotherapeutic experiment

Six-week-old BALB/c male mice (18-20 g) were housed in a specific pathogen-free environment at the Experimental Animal Center of Pusan National University in pressurized, ventilated cages according to institutional regulations. For a nude mouse xenograft chemotherapeutic experiment, each 2 × 10^6^ of uninfected and *P. gingivalis*–infected OSC-20 cells were injected subcutaneously into the left and right back, respectively, of nude mice. Two weeks after injection, the tumor was visible, and then, Taxol treatment was initiated. Taxol, purchased from Sigma-Aldrich (St. Louis, MO), was initially dissolved in 100% DMSO at a concentration of 10 mg/mL and stored at −20 °C. PBS was used as a negative control vehicle. For chemotherapy experiments, 10 mg/kg of Taxol or vehicle was infused intraperitoneally twice a week for 4 weeks. All animal practices in this study were approved and conducted in accordance with the guidelines of the Association of Laboratory Animal Science of Pusan National University (PNU-2015-0893).

### Histology

Tumor mass and lung tissue samples were fixed in 10% neutral-buffered formalin solution and embedded in paraffin. Tissue sections were stained with hematoxylin and eosin (H & E) to examine tissue morphology, and H & E stained sections were observed under light microscopy.

### OSCC metastasis experiment

We injected 2 × 10^6^ OSC-20 cells diluted in 100 μL of PBS into the tail vein of 7-week-old BALB/c male mice after they were housed for 1 week. On the day of the injection, the mice were divided randomly into two groups. The mice were sacrificed after 30 days using Zoletil/Rompun intravenous injection, and the lungs were removed and fixed in 10% formalin. For further analysis, the lung tissue samples were trimmed, processed, embedded, sectioned, and stained with H & E to examine tissue morphology. All mice were fed *ad libitum* and housed in accordance with the guidelines of the Association of Laboratory Animal Science of Pusan National University (PNU-2015-0893).

### Statistical analysis

Statistical analysis was performed with GraphPad Prism 5.03 (GraphPad Software, Inc., La Jolla, CA). Data were analyzed using a Student's *t* test to compare control and *P. gingivalis*–infected groups. All data are presented as mean ± SD. *P* values less than 0.05 were considered significant.

## SUPPLEMENTARY MATERIALS FIGURES



## Abbreviations

OSCC: oral squamous cell carcinoma
CSC: cancer stem cell
NICD: Notch intracellular domain
