# Supplementary material for: Oral cancer cells sustainedly infected with Porphyromonas gingivalis exhibit resistance to Taxol and have higher metastatic potential
Source: Oncotarget. 2017 Mar 24;8(29):46981–92. doi: 10.18632/oncotarget.16550 (PMC5564538; doi:10.18632/oncotarget.16550)
Supplement: Supplementary file 1 [file oncotarget-08-46981-s001.pdf]

Oral cancer cells sustainedly infected with *Porphyromonas gingivalis* exhibit resistance to Taxol and have higher metastatic potential

SUPPLEMENTARY FIGURE

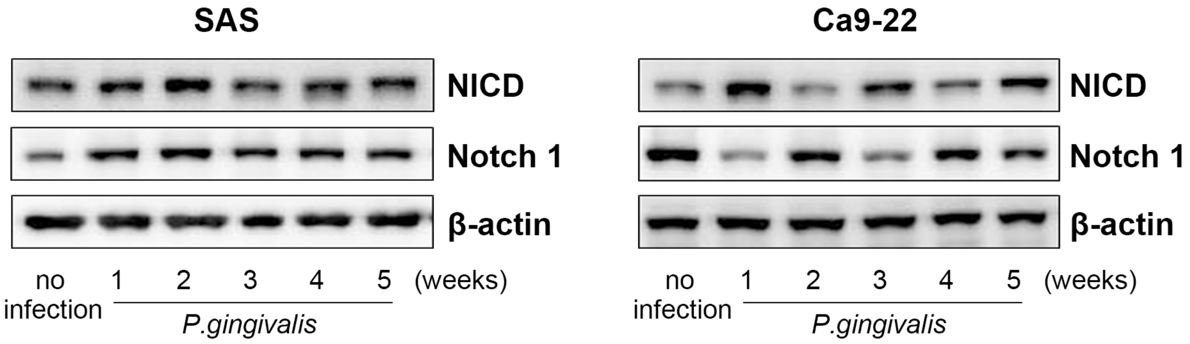

Supplementary Figure 1: Levels of Notch intracellular domain (NICD) were increased in sustainedly *P. gingivalis*–infected OSCC cells. SAS and Ca9-22 OSCC cells were repeatedly infected with *P. gingivalis* twice a week up to five weeks. Expression levels of Notch1 and Notch intracellular domain (NICD) were investigated using a western blot analysis.
